# A Comparative Study of Self-Regulation in Substance Dependent and Non-Dependent Individuals

**DOI:** 10.5539/gjhs.v5n6p40

**Published:** 2013-08-05

**Authors:** Nour Mohammad Bakhshani, Mohsen Hossienbor

**Affiliations:** 1 Children and Adolescents’ Health Research Center, Zahedan University of Medical Sciences, Zahedan, Iran; 2 Department of Psychiatry and Clinical Psychology, Zahedan University of Medical Sciences, Zahedan, Iran

**Keywords:** self-regulation, self-control, substance use, substance dependence, substance abuse

## Abstract

**Background:**

Several factors influence the beginning and maintenance of substance use. The purpose of this study was to examine as well as to compare ‘self-regulation’ in both substance dependent and non-substance dependent individuals.

**Method:**

In a cross-sectional study 228 (118 substance dependent and 110 with no history of using substance) participants aged 16-55 were recruited. All of the participants were asked to complete the Self-Regulation Inventory (SRI-25) and a demographic characteristics data checklist. Data was analyzed using descriptive statistics (frequency, mean and standard deviation) and the t-test.

**Results:**

The results showed significant differences between substance dependent and non- substance dependent groups in all the scales of the self-regulation inventory including positive actions, controllability, expression of feelings and needs, assertiveness, and well-being seeking (p<0.01).

**Conclusion:**

Self-regulation and self-control skills in drug dependent individuals are lower than those without substance dependence individuals. It is concluded that substance use may related to a deficiency in self-control and regulation of feelings. Therefore, for prevention and treatment of substance dependence disorder, it is necessary to work out and exploit strategies that include the improvement of self-regulation.

## 1. Introduction

Addiction is an important worldwide public health problem with various negative effects (Newcomb & Locke, 2005). Two Substance use disorders in DSM-IV are substance abuse and substance dependence. People with substance dependence experience tolerance and withdrawal, but those with substance abuse have maladaptive pattern of substances (American Psychiatric Association, 2000).

It is commonly held that certain individuals are vulnerable to substance dependence/substance abuse. Psychological variables such as attachment style (Kassel, Wardle, & Roberts, 2007) attachment trauma (Padykula & Conklin, 2010) and self-regulation (Percy, 2008) are held to be related to drug use. The problems in the development of attachment and low self-regulation might be related to the vulnerability to develop substance dependence (Khantzian, 2003). Deficiencies in regulating emotions related to drug experimentation and dependence (Dawes, Tarter, & Kirisci, 1997; Khantzian, 1997).

Self-regulation, being stated as an important determinant of psychological adaptability in children, is defined as the competence to regulate the attention, feelings and actions as coordinated with internal and external needs. Self-regulation is an important personality process through which individuals control their thoughts, feelings, impulses, needs and behaviors (Baumeister, Gailliot, DeWall, & Oaten, 2006). Various theoretical models (Abar, Carter, & Winsler, 2009; J. Block & J. H. Block, 1980) argue that the adaptive responses to surrounding challenges are made easier by self-regulation. In earlier studies it was the childhood period that received most of the attention, but more studies having been done on adolescents to support the idea of there being a relationship between self-regulation and the relevant structures (such as positive action and controllability) with adaptability in adolescents. For instance, the children with lower self-control showed greater drug use (J. Block, J. H. Block, & Keyes, 1988; Caspi, Henry, McGee, Moffitt, & Silva, 1995). Low self-regulation and readiness for risk-taking behaviors can also make adolescents more susceptible to the use drugs. A study by Quinn and Fromme found that self-regulation predicted less negative effects of alcohol (2010). High self-regulation buffers the substance use (Neal & Carey, 2007; Wills, Ainette, Stoolmiller, Gibbons, & Shinar, 2008). Also, Pearson et al. confirmed moderating effect of self-regulation on alcohol related problems but not on alcohol consumption (Pearson, D’Lima, & Kelley, 2011).

The negative affect regarded as a provocative factor in using drugs and developing it to a dysfunctional adaptive strategy. The individuals, thus, with low self-regulation are susceptible to use drugs as a coping method, because they lack sufficient skills to regulate their emotions and, as a result, they rely on external factors (Diaz & Fruhauf, 1991). Consequently, the adolescents lower in controlling their impulses experience a higher level of using drugs. Block et al. (1988), Baumeister et al. (2007) and Magar et al. (2008) have reported a significant relationship between low self-control competence and drug use by boys in the six months later. Although, there is a wealth of information on self-regulation and drug use but Most of the literature refers to children and adolescents. The current study mainly was based on adults and designed to examine the status of self-regulation ability among Iranian addicted (substance dependent) and non-addicted persons.

## 2. Method

In this cross-sectional study, data were obtained from 228 participants aged 16-55 years, 118 individuals with substance dependence were recruited randomly from those coming to an addiction treatment clinic of Baharan hospital (a university psychiatric center and 110 individuals with no history of substance use were selected from those accompanying the patients, students, and staff. Table 1 shows the frequency of the participants in the study according to the gender and age.

**Table 1:** Demographic characteristics of participants

Group	Gender		Age		

	Male (F %)	Female (F %)	Mean (SD)	Min	Max
Dependent	97(82.2)	21(17.8)	29.43(7.81)	16	55
Non-dependent	55(50)	55(50)	27.99(8.54)	17	50

For data gathering we used the Self-Regulation Inventory (SRI-25). The SRI designed by Grossarth, Maticek and Eysenk in 1995 to assess health-related oppositional behaviors and its short 25-item version was introduced by Ibanez et al in 2005. This inventory measures the self-regulation capacity in five areas including positive action, controllability, expression of feelings and needs, assertiveness and well-being seeking (satisfaction with oneself and others) (Ibáñez, Ruipérez, Moya, Marqués, & Ortet, 2005). Scores for the scales ranges between a minimum of 25 and a maximum of 150. Higher scores indicate higher levels of self-regulation and the skills related to it (such as positive action, controllability, expression of feelings and needs or well-being seeking). There is a reliable and valid Persian (Iranian) form of this inventory (Ghaleban & Besharat, 2011). In order to examine the consistency of the self-regulation inventory, the exploratory factor analysis through the principle component method was used. Five factors were extracted: positive action, controllability, expression of feelings and needs, assertiveness and well-being seeking in order to measure the internal consistency, the Cronbach Alpha test was relied upon and that the coefficients for the 357-member sample were 0.93, 0.87, 0.91, 0.92, and 0.90 showing satisfactory internal reliability (Besharat, 2011).

The data was analyzed by means of the SPSS software, using descriptive statistics (mean and standard deviation) and t-test in order to compare the score means of the two dependent and non-dependent groups.

## 3. Results

The results of comparing the score means of self-regulation between dependent and non-dependent groups showed that the dependent individuals had lower scores than non-dependent individuals. Differences between two groups across the subscales of SRI were significant. Table 2 provides the results of t-test for comparing the mean scores of substance dependent and normal individual on five subscales of SRI.

**Table 2:** The means, standard deviations and results of t-test on five subscale of self-regulation inventory in dependent and non-dependent groups

Self-regulation Subscale	Group	Mean	Standard Deviation	t	P
Positive Action	Dependent	21.05	4.46	- 3.35	0.001
	Non-dependent	22.89	3.71		
Controllability	Dependent	18.39	4.27	- 8.90	0.000
	Non-dependent	22.88	3.20		
Expression of Feelings and Needs	Dependent	11.19	2.59	3.14	0.002
	Non-dependent	10.23	1.92		
Assertiveness	Dependent	18.00	5.57	- 5.17	0.000
	Non-dependent	21.44	4.37		
Well-being Seeking	Dependent	20.81	6.83	- 6.80	0.000
	Non-dependent	25.74	3.44		

The results of t-test on means scores of men and women in substance dependent group show no significant differences (Table 3). Additionally, meaningful differences were observed between non-dependent men and women (Table 4).

**Table 3:** The means, standard deviations and results of t-test on self-regulation subscales in dependent men and women

Self-regulation subscales	Gender	N	Mean	Standard deviation	t	P
Positive action	Male	97	21.25	4.56	1.03	0.3
	Female	21	20.14	3.92		
Controllability	Male	97	18.46	4.90	0.35	0.7
	Female	21	18.09	5.16		
Expression of feelings and needs	Male	97	11.19	2.57	0.009	0.9
	female	21	11.19	2.78		
Assertiveness	Male	97	18.31	5.50	1.34	0.1
	Female	21	16.52	5.78		
Well-being seeking	Male	97	20.69	6.73	0.418	0.6
	Female	21	21.38	7.44		

**Table 4:** The means, standard deviations and results of t-test on self-regulation subscales in non-dependent participants

Self-regulation subscales	Gender	N	Mean	Standard deviation	t	P
Positive action	Male	55	23.77	3.60	2.41	0.01
	Female	55	22.05	3.65		
Controllability	Male	55	23.60	3.15	2.40	0.01
	Female	55	22.16	3.11		
Expression of feelings and needs	Male	55	10.60	1.78	2.00	0.04
	Female	55	9.87	2.00		
Assertiveness	Male	55	22.56	4.17	2.76	0.007
	Female	55	20.32	4.31		
Well-being seeking	Male	55	26.43	3.00	2.13	0.03
	Female	55	25.05	3.73		

## 4. Discussion

As the results of the study showed, there were significant differences between the dependent and non-dependent participants in all the subscales of self-regulation. The current findings are in accordance with the results of the study done by Besharat and Ghal’eban (2011). They found that the self-regulation in the drug users is lower than non-users, that is, they reported that a deficiency in controllability and regulating of affect is most likely related to drug abuse. Also Oleary et al. (1992) found that individuals having lower self-regulation skills are more likely to use drugs and to show high-risk behaviors. The results of the current study illustrated that the dependent individuals have less “controllability” in turn possibly making them more vulnerable to drug abuse. It can be hypothesized that having a sense of self-efficiency, emotional independence, and having skills of active regulation play a decisive role in protecting individuals from high-risk behaviors such as drug abuse. Special attention however has been paid by many scientists to this issue (Bandura, 1991; O’leary et al., 1992). Dependent individuals show lower self-efficiency beliefs and this can be detrimental to psychological health and may facilitate drug use/abuse. Other studies have pointed to the role of the individuals’ beliefs in being able to end or to decrease negative emotions as a mechanism of self-regulation and to prevent drug abuse (Annis & Davies, 1988; Baumeister et al., 2007; Condiotte & Lichtenstein, 1981). These beliefs differ from person to person and are in direct relationship with different coping skills or reducing negative emotions (Thorberg & Lyvers, 2006) and can affect several aspects of the life such as goals setting, decision-making, and persistent of efforts when encounter to challenging situation (Bandura, 1991, 1993; Pintrich & Degrroot 1990; Walton & Roberts, 2004).

Self-regulation and self-control beliefs reduce both the probability of the development of disorders and the treatment process of various disorders (O’leary et al., 1992). The above-mentioned beliefs, also, improve the function of the body’s immune system through the modulation of stress (Grossarth-Maticek et al., 1999; Marqués et al., 2005; O’leary et al., 1992); the autonomy and impulse-control capacity (Penley & Tomaka, 2002; Vohs, Baumeister, & Ciarocco, 2005). Moreover, low self-regulation and self-control have a close relationship with behaviors like smoking, alcohol consumption, drug use, and behaviors threatening one’s health.

A study done by Baumeister and Vohs (2007) on self-regulation of patients reliant on drugs indicated that dependent individuals show lower self-regulation than non-dependent ones, and that “incomplete” regulation and “failure” in complete self-control or false regulation (a control that not leading to desired results) make the individuals susceptible to use drugs. In addition, O’leary et al. (1992) found that self-regulation beliefs are related to different behaviors like smoking, drinking alcohol, and preventive strategy of using drugs.

In summary, deficit in regulation (failure in self-control) or false regulation (a control leading to undesired results) makes the individuals susceptible to use drugs (Baumeister et al., 2007; Ghaleban & Besharat, 2011; Ibáñez et al., 2005). However deficits in self- regulation may occur due to various factors including bio psychosocial variables. For example, Besharat and Ghal’eban (2011) have pointed to the main role of frontal cortex in the emotional-regulation and its relationship with the problems of using drugs. It seems, therefore, highly essential for the researchers, policy makers, and health-care experts to consider the self-regulation competence and its interaction with the internal and external condition in prevention and treatment plans. Undoubtedly, integrating the individual, family, cultural and social factors in prevention and treatment strategies will increase the effectiveness of these programs.

Using Convenience sampling method for recruiting individuals without substance dependence is a limitation of our study. In spite of advantages of this method, such as availability and quickness of gathering participants, but caution must be taken when generalizing the results of current study because sample might not representative the study population.
